# Cx43-hemichannel function and regulation in physiology and pathophysiology: insights from the bovine corneal endothelial cell system and beyond

**DOI:** 10.3389/fphys.2014.00348

**Published:** 2014-09-12

**Authors:** Catheleyne D'hondt, Jegan Iyyathurai, Bernard Himpens, Luc Leybaert, Geert Bultynck

**Affiliations:** ^1^Laboratory of Molecular and Cellular Signaling, Department of Cellular and Molecular Medicine, Katholieke Universiteit LeuvenLeuven, Belgium; ^2^Physiology Group, Department of Basic Medical Sciences, Faculty of Medicine and Health Sciences, Ghent UniversityGhent, Belgium

**Keywords:** intercellular communication, hemichannels, loop/tail interactions, actin/myosin contractility, selective inhibition of Cx43 hemichannels, corneal endothelial cells

## Abstract

Intercellular communication in primary bovine corneal endothelial cells (BCECs) is mainly driven by the release of extracellular ATP through Cx43 hemichannels. Studying the characteristics of Ca^2+^-wave propagation in BCECs, an important form of intercellular communication, in response to physiological signaling events has led to the discovery of important insights in the functional properties and regulation of native Cx43 hemichannels. Together with ectopic expression models for Cx43 hemichannels and truncated/mutated Cx43 versions, it became very clear that loop/tail interactions play a key role in controlling the activity of Cx43 hemichannels. Interestingly, the negative regulation of Cx43 hemichannels by enhanced actin/myosin contractility seems to impinge upon loss of these loop/tail interactions essential for opening Cx43 hemichannels. Finally, these molecular insights have spurred the development of novel peptide tools that can selectively inhibit Cx43 hemichannels, but neither Cx43 gap junctions nor hemichannels formed by other Cx isoforms. These tools now set the stage to hunt for novel physiological functions for Cx43 hemichannels in primary cells and tissues and to tackle disease conditions associated with excessive, pathological Cx43-hemichannel openings.

## Properties of the bovine cornea with focus on the endothelial cell layer

### Morphological properties

**Figure 1:**
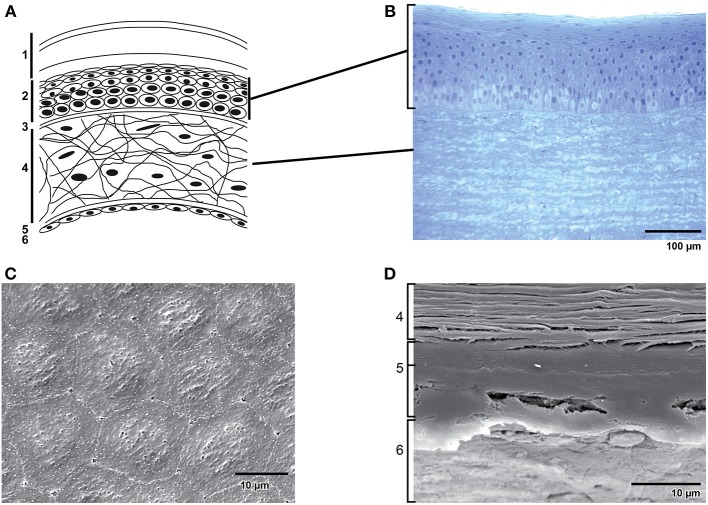
**Structure of the cornea**. **(A)** Schematic representation of the structure of the human cornea: 1. Tear film with (starting from the outermost surface) a lipid layer, aqueous layer and a mucous layer. 2. Epithelial cells. 3. Bowman's membrane. 4. Stroma with keratocytes in an extracellular matrix of collagen fibrils and glycoaminoglycans. 5. Descemet's membrane. 6. Endothelial monolayer. **(B)** Transverse section of the epithelium and part of the stroma in bovine cornea. **(C)** Scanning electron microscopy of bovine corneal endothelium. Note the hexagonal shape of the cells. **(D)** Scanning electron microscopy of a transverse section of bovine cornea showing (from bottom to top) endothelium (6), the two distinct layers of Descemet's membrane (5) and a part of the stroma (4).

The cornea is a transparent, convex, avascular continuation of the sclera covering the front part of the eye. Light enters the eye through the cornea, which provides about 2/3 of the eye's refractive power and forms together with the lens the focusing power of the eye (Maurice, 1957). At the posterior side of the cornea lies the anterior segment, which contains aqueous humor. The delicate balance between the production and absorption of aqueous humor keeps the anterior chamber pressurized (~20 mm Hg) contributing to the maintenance of the curvature of the cornea. When this balance is disturbed glaucoma can occur (Tandon and Autar, 1989). Unlike most tissues in the body, the cornea is avascular; it contains no blood vessels to nourish it or to protect it against infection. Instead, tiny vessels at the outermost edge of the cornea along with the tears and aqueous humor take care of cell nourishment. The adult human cornea is ~0.5 millimeter thick and is arranged in five basic layers: the stratified epithelium, Bowman's membrane, stroma, Descemet's membrane and the endothelium (Figure 1A). The bovine cornea is thicker than the human cornea due to a thicker stroma and more epithelial cell layers (Figure 1B). The endothelium is the innermost layer of the cornea, located just underneath Descemet's membrane, and forms an interface between the stroma and anterior chamber. The corneal endothelium is a 4–6 μm thick, non-regenerative monolayer of polygonal-shaped, mostly hexagonal cells (Figures 1C,D) with a diameter of about 20 μm (Joyce, 2003). The human corneal endothelium consists of about 400,000 cells. On the apical cell surface facing the anterior chamber, numerous small microvilli are present, and extensive interdigitations appear on lateral and basal plasma membranes. A circumferential band of actin filaments, located toward the apical aspect of the cells, helps to maintain cell shape and mediates cellular migration. The corneal endothelial monolayer is able to persist thanks to the complex interplay between the extracellular matrix, integrins, interendothelial junction proteins and the actin cytoskeleton. As in vascular endothelium, corneal endothelial cells are interconnected by a complex set of interendothelial junction proteins that comprise tight junctions, adherens junctions and gap junctions. Whereas gap junctions form plaques of transmembrane channels between adjacent cells, tight junctions form a belt around the apical pole of a polar cell making a tight connection with the belt around the neighboring cells and adherens junctions form pericellular zipperlike structures along the cell border through their transmembrane homophilic adhesion (for review see Mehta and Malik, 2006).

### Physiological properties

The cornea with its smooth, transparent, strong and durable characteristics has several functions in the eye. The transparent convex surface of the cornea acts as the eye's outermost optical element of fixed focal length and enables focusing light onto the retina of the eye, hereby making the cornea act as an important lens. The avascular nature of the cornea and the regular arrangement of the collagen fibrils of the stroma ensure the transparency of the cornea (Davson, 1949; Dikstein and Maurice, 1972; Maurice, 1972; Hull et al., 1977; Riley, 1985; Bonanno and Giasson, 1992a,b; Bonanno, 2003), which is crucial for good vision. The cornea also serves as a filter, screening out some of the most damaging ultraviolet (UV) wavelengths in sunlight. Without this protection, the lens and the retina would be highly susceptible to injury from UV radiation. The cornea also functions as a barrier against influx of solutes, dust, germs, pathogens, and other injurious matter, into the eye. This protective task is primarily carried out by the epithelium but is shared with the eyelids, the eye socket, tears, and the sclera. Bowman's membrane and Descemet's membrane serve as a protective barrier against infection and injuries (Beuerman and Pedroza, 1996). The corneal epithelium, with its microvilli and microplicae, acts as a mucous surface that absorbs oxygen and nutrients from tears. The endothelium absorbs nutrients from the aqueous humor in order to provide the rest of the cornea of these nutrients.

The main physiological role of the corneal endothelium is maintenance of corneal transparency by controlling stromal hydration (Davson, 1949; Dikstein and Maurice, 1972; Maurice, 1972; Hull et al., 1977; Riley, 1985; Bonanno and Giasson, 1992a,b; Bonanno, 2003). Since the paracellular space between the corneal endothelial cells is partially occluded by the discontinuous focal adhesion tight junctions and the sinuous interdigitations of lateral membranes of adjacent cells, the endothelial barrier is leaky and permits paracellular passage of fluid and nutrients from the aqueous humor into the avascular cornea. The endothelium counteracts the tendency of the corneal stroma to swell by removing excess of stromal fluid and by regulating paracellular permeability through a complex interplay of cellular adhesive forces balanced against counter-adhesive forces generated by actomyosin molecular motors and through an active fluid transport from the stroma into the anterior chamber of the eye (reviewed in Bonanno, 2003). Both the “pump” and barrier functions of the endothelium are essential for maintaining the relatively dehydrated state of the stroma required for transparency. This continuous maintenance of equilibrium is referred to as the pump-leak mechanism (Davson, 1949; Maurice, 1972).

### Corneal dysfunction

#### Regulation of barrier integrity

In order to maintain corneal function, regulation of the barrier integrity is crucial. The barrier function of the endothelial monolayer is controlled by the activation of different signaling mechanisms that affect paracellular and transcellular pathways. Although much is known about the identity of ion transport mechanisms in the corneal endothelium (Bonanno, 2003), the mechanisms of cell signaling that regulate barrier integrity are just beginning to be understood (Riley et al., 1996, 1998; Bonanno, 2003). An increase in contractility of the cortical perijunctional actomyosin ring (PAMR) induces a centripetal force that opposes the tethering forces at tight junctions and adherens junctions and results in a breakdown of the endothelial barrier integrity (i.e., an increase in paracellular permeability) (Garcia and Schaphorst, 1995; Turner et al., 1997; Stevenson, 1999; Turner, 2000). The contractility of the actin cytoskeleton, including that of PAMR, is regulated through actomyosin interaction that is induced by the phosphorylation of the regulatory light chain of myosin II, also called myosin light chain (MLC) (Somlyo and Somlyo, 2000; Kamm and Stull, 2001). Phosphorylation of MLC, influenced by MLCK, PKC, or Rho kinase, induces an altered contractility of the actin cytoskeleton (Dudek and Garcia, 2001; Bogatcheva et al., 2002; Satpathy et al., 2004), which results in a significant breakdown of barrier integrity and the formation of interendothelial gaps (Garcia et al., 1995; Zhao and Davis, 1999; Van Nieuw Amerongen et al., 2000; Vouret-Craviari et al., 2002). Restoration of barrier integrity mainly occurs through relaxation of the actin cytoskeleton as a result of a decreased MLC phosphorylation via PKA-mediated inactivation of MLCK (Garcia et al., 1997; Somlyo and Somlyo, 2000; Kamm and Stull, 2001). In BCEC, barrier integrity is controlled via MLCK-, PKC- and Rho kinase-mediated pathways that regulate the MLC phosphorylation status (Satpathy et al., 2004, 2005; Srinivas et al., 2004).

#### Cell loss

In humans, corneal endothelial cell count averages around 3000 (Waring et al., 1982) to 6000 (Bourne, 2003) cells/mm^2^ at birth and then slowly declines with age (Bourne and Brubaker, 1982, 1983; Bourne et al., 1997). With advancing age corneal endothelial cells also display greater morphological heterogeneity, a smaller percentage of hexagonal cells (Bourne and Brubaker, 1983; Bourne, 2003), and a decreased endothelial cell density and corneal thickness (Armitage et al., 2003; Zhu and Joyce, 2004; Mimura and Joyce, 2006) (for review see Moller-Pedersen, 1997; Bourne, 2001; Joyce, 2003). This cell loss can be accelerated by damage (Bourne and Brubaker, 1982, 1983; Bourne et al., 1997), pathological factors, such as primary corneal endotheliopathies (Schultz et al., 1984; Gagnon et al., 1997) (e.g., Fuchs's dystrophy), inflammation, glaucoma, prolonged UV exposure, a number of drugs and mechanical injury during intraocular surgery (Rao et al., 1978; Brooks and Gillies, 1991; Armitage et al., 2003) or laser procedures (Bergmann et al., 1991).

In the healthy adult cornea, endothelial cell count is between 2000 and 2500 cells/mm^2^. When this cell count falls below a critical level (500–1000 cells/mm^2^) endothelial dysfunction occurs (Edelhauser, 2000; Bonanno, 2003; Bourne, 2003; Bourne and McLaren, 2004; Bourne et al., 2004). Loss of an intact endothelial monolayer induces corneal edema, which causes the cornea to become cloudy, resulting in a loss of visual acuity (Riley, 1985; Landshman et al., 1988; Tuft and Coster, 1990; Riley et al., 1998; George and Larkin, 2004). In this situation, corneal transplantation is required to restore a functional endothelium (George and Larkin, 2004) as there are no pharmacological approaches to overcome endothelial dysfunction.

#### Wound repair

In many cell types, including corneal epithelium, cell division contributes to wound repair. However, evidence strongly suggests that cell division, if it occurs, plays only a minor role as a repair mechanism in mature corneal endothelium *in vivo*. Numerous studies indicate that wound healing in mature corneal endothelium *in vivo* mainly occurs by cell enlargement, in which flattening of the cells occurs, and cell migration, in which individual cells move from the wound edge to repopulate the wound (for review see Joyce, 2003). As a consequence of this form of repair, there is not only an age-related increase in cell size (polymegathism) of corneal endothelial cells, but also a gradual alteration from the typical hexagonal shape to a more polygonal, and finally pleomorphic shape (pleomorphism) (Laing et al., 1976; Matsubara and Tanishima, 1983; Murphy et al., 1984; Hoppenreijs et al., 1992). Stimulation of the cAMP pathway enhances individual cell migration, and prostaglandin E2 (PGE2) may be an endogenous stimulator of this response during corneal endothelial wound repair (Joyce and Meklir, 1994).

When only a small number of cells have been injured, healing occurs solely by enlargement of cells immediately adjacent to the wound. Repair appears to be initiated by membrane ruffling into the wound area. Once cells have made contact with each other, they stop ruffling and establish mature cell-cell contacts (for review see Joyce, 2003). In large wounds, repair occurs mainly as the result of coordinated enlargement and migration of cells adjacent to the wound and a few rows behind the wound edge. Cells initially enlarge and elongate into the wound area, causing movement of the monolayer as a sheet to cover the wound. Without losing contact with neighboring cells, the enlarged cells subsequently contract and pull surrounding cells into the wound area (Ichijima et al., 1993a,b). This form of repair has been designated as monolayer “spreading” (Joyce et al., 1989). Both monolayer spreading and cell migration result, at least in part, from alterations in the expression and organization of cytoskeletal elements, such as actin filaments and microtubules.

## Intercellular communication in bovine corneal endothelial cell monolayers

### Connexin and pannexin expression profile

**Figure 2:**
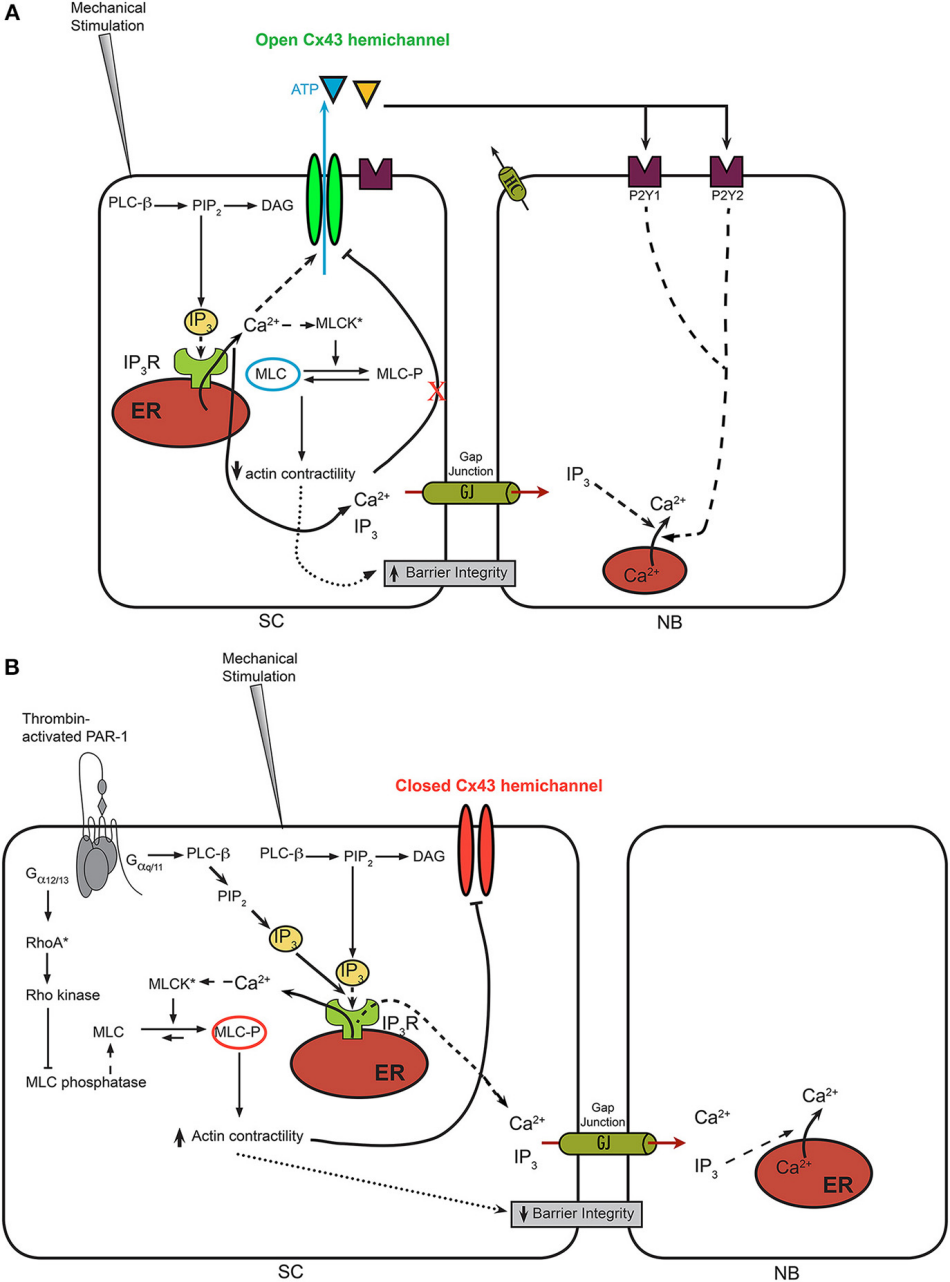
**A model for Ca^2+^-wave propagation in BCECs in normal conditions vs. thrombin-treated conditions. (A)** In normal BCECs, it is hypothesized that mechanical stimulation leads to a moderate rise in cytosolic [Ca^2+^] via IP_3_-dependent signaling mechanisms, which leads to the opening of Cx43 hemichannels and the flux of ATP from the cytosol into the extracellular environment. This allows the propagation of the Ca^2+^ from the “stimulated cell” (SC) to neighboring (NB) cells via activation of purinergic receptor and downstream IP_3_-induced Ca^2+^ signaling. Under these conditions, it is anticipated that MLC remains in a dephosphorylated state, because of excess MLC phosphatase activity despite the potential activation of MLC kinases through Ca^2+^/CaM. **(B)** In thrombin-pre-treated BCECs, the concerted activation of IP_3_/Ca^2+^ signaling, leading to the activation MLC kinase combined with the activation RhoA/Rho kinase, leading to the inhibition of MLC phosphatase, leads to an enhanced contractility of the actin/myosin cytoskeleton. The latter seems linked to the Cx43 hemichannels, likely through its C-terminal tail. Increased actin/myosin contractility is proposed to displace the C-terminal tail from the cytoplasmic loop, thereby annihilating loop/tail interactions essential for Cx43-hemichannel opening. As such, Ca^2+^ signaling triggered by mechanical stimulation will not be able to lead to opening of these “locked”/'closed” Cx43 hemichannels, preventing extracellular ATP release and suppressing ATP-driven Ca^2+^-wave propagation to neighboring cells. In addition to the effects of the actin/myosin contractility on Cx43 hemichannels, actin/myosin contractility may alter tight junctions and the ability of “head-to-head” docked hemichannels to form gap junction channels.

Intercellular communication between bovine corneal endothelial cells typically has been studied using a local mechanically induced stimulus applied to a single cell. Communication between cells can be easily monitored by measuring intercellular Ca^2+^-wave propagation within the BCEC monolayer that is loaded with a fluorescent Ca^2+^ dye, Fluo-4. A detailed description and visualized representation of the method can be found elsewhere (D'hondt et al., 2013a). The focus on Ca^2+^ signaling in this type of experiments is due to the fact that mechanical stimulation elicits an increase in cytosolic [Ca^2+^], involving several Ca^2+^-flux mechanisms, including the inositol 1,4,5-trisphosphate (IP_3_) receptor (IP_3_R)-mediated release of Ca^2+^ from the endoplasmic reticulum due to the activation of phospholipase C (PLC) (Leybaert and Sanderson, 2012; D'hondt et al., 2013a) (Figure 2A). A detailed analysis of the spatio-temporal activation of PLC upon mechanical stimulation has been described by others in MDCK cells (Tsukamoto et al., 2010), although the exact mechanisms underlying this PLC activation remain elusive. Importantly, the properties of the intercellular Ca^2+^ wave, including the speed of propagation and the extent of propagation (i.e., active area, corresponding to the area of cells responding with a cytosolic [Ca^2+^] rise) provide an invaluable tool to analyze the properties of communicating channels, like connexins and pannexins (Leybaert and Sanderson, 2012). Furthermore, the occurrence of intercellular Ca^2+^ waves has been described in a variety of cell systems and tissues, including the retina, cochlea, blood vessels, brain, liver with important physiological functions and pathophysiological consequences (Leybaert and Sanderson, 2012). Furthermore, the method impinges on the endogenously expressed channels and receptors and physiological signaling molecules present within the primary cell system. In particular, the presence of different connexin isoforms, including Cx26, Cx30.3, Cx31, Cx32, Cx36, Cx43, Cx45, Cx46, Cx46.6, and Cx50 and pannexin isoforms, including Panx1, Panx2, and Panx3, has been confirmed at the mRNA level (Ponsaerts et al., 2010a). At the protein level, the presence of Cx43 in BCEC lysates could be demonstrated via immunoblotting (Ponsaerts et al., 2010b), while immunocytochemistry showed the presence of Cx26 and Cx43 between corneal endothelial cells (Laux-Fenton et al., 2003). While these observations do not allow making conclusions about the relative expression of Cx/Panx isoforms or about the predominant Cx/Pan isoforms, the presence of a plethora of Cx/Panx isoforms do underpin that BCECs are very well suited for studying intercellular communication. Furthermore, these aspects are not limited to BCECs, since human corneal endothelial cells also express connexin isoforms, including Cx43, and display intercellular communication (Williams and Watsky, 2002). The upregulation of Cx43 at the protein level seems an important marker for the assessment of novel strategies to improve the preservation and maintenance of functional human donor cornea. In particular, the treatment with vasoactive intestinal peptide and ciliary neurotrophic factor, endogenous autocrine molecules from corneal endothelial cells, seem to be very promising agents to promote the differentiation and survival of corneal endothelial cells (Koh et al., 2011; Koh, 2012). Finally, the presence of connexins in corneal endothelial cells also seems to prevent their proliferation. As such, Cx43 knockdown has been implicated as a novel therapeutic strategy to accelerate wound healing in the corneal endothelium (Nakano et al., 2008) and to promote re-epithelialization by suppressing stromal oedema and inflammatory responses (Grupcheva et al., 2012).

### Paracrine signaling

**Figure 3:**
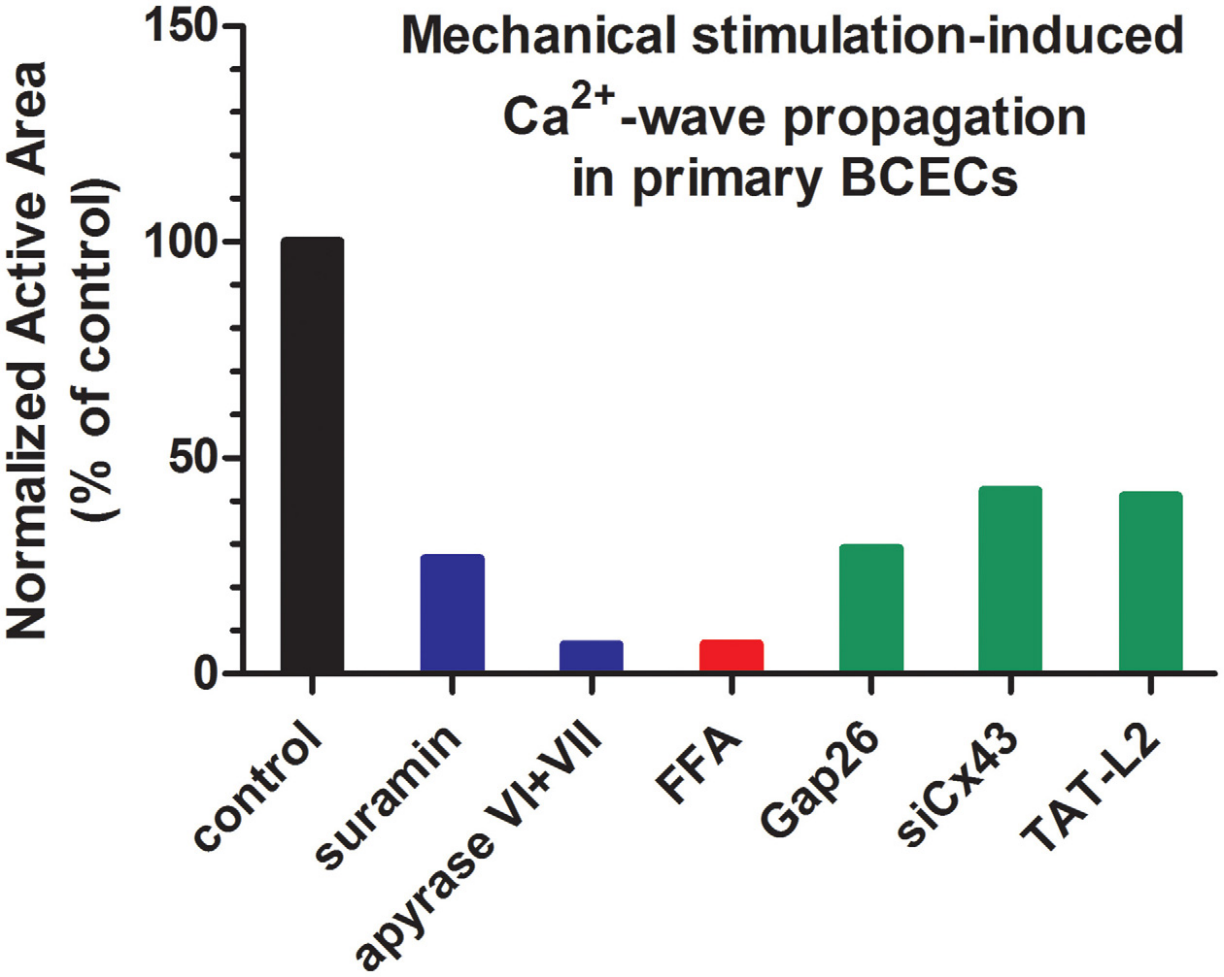
**Characteristics of intercellular communication in BCECs**. A graph depicting the characteristics of intercellular communication in BCECs, based on mechanical stimulation-induced Ca^2+^-wave propagation data (active area) previously published in (Gomes et al., 2005a,b; Ponsaerts et al., 2010b). Data were further normalized to their respective controls (set at 100%). The graph is intended to indicate the relevance of ATP release (blue bars), hemichannels (red bar) and Cx43-based hemichannels (green bars). In general, the data indicate that mechanical stimulation-induced Ca^2+^ wave propagation is almost completely driven by release of ATP into the extracellular environment (~90% inhibition by ATP-degrading enzymes) and that Cx43 hemichannels are a major release pathway for this ATP (~60% inhibition upon Cx43 knockdown or inhibition), although other Cx and/or Panx isoforms likely contribute to ATP release. Since this graph is intended to provide a general view, readers should access the original research paper for obtaining information about the mean data and their respective SEM values.

Intercellular communication, including intercellular Ca^2+^-wave propagation, can occur via two main pathways: (i) direct coupling via gap junction channel, allowing the passage of signaling molecules that can mobilize intracellular Ca^2+^, like IP_3_, and (ii) indirect coupling via hemichannels, allowing the release of signaling molecules, like ATP, that can trigger intracellular Ca^2+^ signaling by acting on ionotropic and/or metabotropic receptors, like the P2X and P2Y purinergic receptors (Leybaert and Sanderson, 2012) (Figure 2A). In BCECs, a prominent role for paracrine signaling via extracellular ATP was found as the main driving mechanism for intercellular Ca^2+^-wave propagation in response to mechanical stimulation (Gomes et al., 2005b), although gap junctions are present and are operative in BCECs (Gomes et al., 2006). Indeed, exogenous application of the apyrase VI + VII cocktail dramatically diminished the active area of the intercellular Ca^2+^-wave (Figure 3), while inhibiting endogenously expressed ectonucleotidases using exogenously applied inhibitors, like ARL-67156, dramatically increased the intercellular communication (Gomes et al., 2005b). These observations were supported by direct luciferin/luciferase bioluminescent measurements of the extracellular ATP levels, showing that mechanical stimulation induced ATP release in the extracellular environment. Extracellular ATP-driven intercellular Ca^2+^-wave propagation requires the presence of ATP receptors that initiate Ca^2+^ signaling upon activation. At the mRNA level, different ionotropic P2X receptors, including P2X3, P2X4, P2X5, and P2X7 and different metabotropic P2Y receptors, including P2Y1, P2Y2, P2Y4, P2Y6, P2Y10, and P2Y12 are expressed (Gomes et al., 2005b; Ponsaerts et al., 2010a), correlating with previous functional evidence (Srinivas et al., 1998). Also mRNA of the ectonucleotidases, CD39 and CD73, was detected, confirming the presence of ATP-degrading enzymes. The activity of ectonucleotidases seems to critically control this ATP-driven communication, since aged BCECs display increased ectonucleotidase activity and concomittant reduced ATP-dependent intercellular communication (D'hondt et al., 2009). Consistent with the presence of the purinergic receptors, exogenously added ATP using concentrations in the (sub)μM range was able to induce intracellular Ca^2+^ release with an EC50 of ~1.5 μM. Particularly, P2Y receptors might be responsible for the ATP-induced Ca^2+^ rises and ATP-driven Ca^2+^-wave propagations, since suramin, a non-selective P2X and P2Y antagonist, dramatically reduced intercellular Ca^2+^-wave propagation (Gomes et al., 2005b) (Figure 3). However, the latter result should be interpreted with caution, since a recent report indicated that suramin could also suppress Cx43-hemichannel activity and its associated membrane permeability (Chi et al., 2014).

### Cx43 hemichannels

An important mechanism responsible for the release of ATP seems to be mediated via hemichannels (Gomes et al., 2005a). Similar to the application of exogenous apyrases, the presence of flufenamic acid (FFA), a known hemichannel inhibitor, strongly reduced mechanical stimulation-induced Ca^2+^-wave propagation in BCECs and almost completely blunted mechanical stimulation-induced ATP release without affecting gap junction coupling (Gomes et al., 2005a) (Figure 3). Yet, data obtained using FFA have to be interpreted with caution, since FFA is a broad spectrum ion channel modulator, impacting a variety of non-selective cation channels and K^+^, Ca^2+^, Na^+^ and Cl^−^-permeable channels (see Guinamard et al., 2013 for a detailed discussion). Consistent with the prominent expression of Cx43 in BCECs and the relevance of Cx43 as one of the major Cx isoforms responsible for intercellular communication and intercellular Ca^2+^ waves, Gap26, a Cx43-mimetic peptide, suppressed intercellular communication and ATP release (Gomes et al., 2005a) (Figure 3). It is important to note that Gap26 can also inhibit gap junction-mediated intercellular communication. However, as shown in a recent electrophysiological study, the kinetics for inhibition of hemichannels vs. gap junctions by Gap26 seems to be very different. Indeed, short-term incubations (~5 min) were sufficient to readily inhibit Cx43-hemichannel currents, while long-term incubations (~30 min) were required to inhibit Cx43 gap junction-mediated electrical coupling in cell pairs (Desplantez et al., 2012). The inhibition of Cx43 hemichannels by Gap26 has been further characterized, displaying a right-ward shift of about 20–30 mV for the activation of Cx43 hemichannel currents, suppressing Cx43-hemichannel currents potentiated by physiological elevations in intracellular [Ca^2+^], and inhibiting Cx43-hemichannel currents with an IC50 of about 80 μM (Wang et al., 2012, 2013a). Not only ectopically expressed Cx43 hemichannels could be inhibited, but Gap26 also suppressed native Cx43 hemichannels from pig ventricular cardiomyocytes (Wang et al., 2012). In BCECs, Gap26 applications that are able to inhibit Cx43 hemichannels did not impair gap junctional coupling in fluorescence recovery after photobleaching (FRAP) using carboxyfluorescein (Gomes et al., 2005a). More recently, siRNA-based knock down approaches were applied to directly assess the contribution of Cx43 in this process (Ponsaerts et al., 2010b) (Figure 3). Reducing the endogenous Cx43-protein levels by about 90% resulted in a more than 70% reduction in the active area of the intercellular Ca^2+^ wave in response to mechanical stimulation. Also using Ca^2+^-free solution (EGTA) as a trigger for the opening of hemichannels, the knockdown of Cx43 resulted in a more than 60% reduction in EGTA-induced ATP release. Collectively, these data indicate Cx43 as the main connexin isoform responsible for forming hemichannels and for mediating hemichannel-mediated ATP release in BCECs, thereby driving intercellular Ca^2+^-wave propagation.

## Bovine corneal endothelial cell monolayers as a primary cell model for the study of Cx43-hemichannel properties and regulation

### Cx43 hemichannels as integrators of signaling processes

Cx43 hemichannels can be activated via membrane depolarizations, decreases in extracellular [Ca^2+^] and moderate increases in intracellular [Ca^2+^] (Wang et al., 2013a,c). While strong membrane depolarization (above +50 mV) are needed for the opening of Cx43 hemichannels, increases in cytosolic [Ca^2+^] in the physiological range of 100–500 nM provoke a left-ward shift in the voltage-dependent opening of Cx43 hemichannels (Wang et al., 2012). In this study, moderate intracellular [Ca^2+^] elevations could induce Cx43-hemichannel opening in response to a membrane depolarization in the order of +30 mV, thus shifting the threshold for voltage activation toward the physiological range (Wang et al., 2012). Furthermore, increasing the cytosolic [Ca^2+^] to about 500 nM seems sufficient to open Cx43 hemichannels, allowing the flux of ions and of signaling molecules like ATP (De Vuyst et al., 2009). In that sense, it is not surprising that mechanical stimulation, which triggers cytosolic Ca^2+^ rises, leads to Cx43 hemichannel opening. Furthermore, this Cx43-hemichannel opening seems to be strongly influenced by other physiological signaling cascades in response to extracellular factors. For instance, activation of the plasmalemmal thrombin-sensitive PAR-1 receptors using thrombin or the thrombin receptor activating peptide 6 (TRAP6; SFLLRN) inhibited Cx43-hemichannel activity in BCECs in response to mechanical stimulation (D'hondt et al., 2007a) (Figure 2B). The underlying signaling cascades triggered by thrombin exposure involved the concerted activation of myosin light chain kinase (MLCK), Rho kinase and protein kinase C (PKC) (Garcia et al., 1996; D'hondt et al., 2007a) (Figure 2B). Separate inhibition of one of the signaling cascades nearly completely restored Cx43-hemichannel opening in thrombin-treated BCECs. A common denominator of the different signaling pathways initiated by thrombin is that they all contribute to increased myosin light chain (MLC) phosphorylation (Satpathy et al., 2004), an event that results in increased contractility of the actin cytoskeleton in non-muscle cells (Burridge and Chrzanowska-Wodnicka, 1996). The critical role of the MLC phosphorylation status for controlling Cx43-hemichannel activity has been supported by experiments applying adenosine (D'hondt et al., 2007b), which has previously been demonstrated to induce MLC dephosphorylation in BCEC (Srinivas et al., 2004). Extracellularly added adenosine counteracts RhoA activation via a cAMP-dependent mechanism, prevents the inhibition of MLC phosphatase and opposes thrombin-induced MLC phosphorylation (D'hondt et al., 2007b). Interestingly, pre-incubation of the BCECs with adenosine completely alleviated the thrombin-induced inhibition of Cx43 hemichannels. The critical role of RhoA in the downstream signaling cascade initiated by thrombin and cross-talk to Cx43 hemichannels was supported by the use of C3 toxin, a cell-permeable RhoA inhibitor (Ponsaerts et al., 2012a). This compound completely abolished the thrombin-induced inhibition of Cx43 hemichannels in BCECs. Consistent with this, RhoA was rapidly activated in BCECs exposed to thrombin and C3 toxin pre-treatment completely prevented this RhoA activity.

### Cx43-hemichannel opening is negatively regulated by the actin cytoskeleton

Given the prominent role of MLC phosphorylation in controlling the contractility of the actin/myosin cytoskeleton and the functional link between MLC phosphorylation and decreased Cx43 hemichannel activity, the impact of the actin/myosin contractility on Cx43-hemichannel opening was assessed using a myosin II ATPase inhibitor, (-)-blebbistatin (Straight et al., 2003; Ponsaerts et al., 2008). This compound prevents the contraction of the actin/myosin cytoskeleton (Straight et al., 2003; Lucas-Lopez et al., 2008). Importantly, (-)-blebbistatin does not prevent the thrombin-induced increase in the MLC phosphorylation status, indicating that it does not affect the upstream signaling pathways controlling the activity of kinases or phosphatases that impact the MLC phosphorylation status (Ponsaerts et al., 2008). Strikingly, (-)-blebbistatin completely prevented the thrombin-induced inhibition of Cx43 hemichannel activity in BCECs brought about either mechanical stimulation or by Ca^2+^-free solution. These data indicated that activation of the actomyosin contractile system serves as an endogenous brake counteracting the opening of Cx43 hemichannels (Ponsaerts et al., 2012b). The negative regulation of Cx43 hemichannels seems not restricted to BCECs, but seems to be a generally present and physiologically relevant mechanism (Ponsaerts et al., 2010b). Indeed, follow-up work in HeLa cells and C6 glioma cells ectopically expressing Cx43 revealed a bell-shaped dependent regulation of Cx43-hemichannel activity in response to cytosolic [Ca^2+^]. Moderate rises in cytosolic [Ca^2+^] in the range of 100–500 nM triggered Cx43-hemichannel opening, while excessive rises in cytosolic [Ca^2+^] above 500 nM and the μM range suppressed Cx43-hemichannel opening (De Vuyst et al., 2009; Ponsaerts et al., 2010b). Interestingly, the inhibition of Cx43-hemichannel opening brought about by high cytosolic [Ca^2+^] rises (triggered by 10 μM A23187, a Ca^2+^ ionophore) could be completely prevented by (-)-blebbistatin (Ponsaerts et al., 2010b). This mechanism may not only occur during steady-state rises in cytosolic [Ca^2+^], but may also occur during physiological agonist-induced Ca^2+^ signaling (De Bock et al., 2012b). In recent work, it was shown that bradykinin-triggered Ca^2+^ oscillations in the MDCK epithelial cell line led to the Ca^2+^-dependent opening of Cx43 hemichannels (De Bock et al., 2012b). Interestingly, the opening of Cx43 hemichannels provided a non-selective Ca^2+^-entry pathway and led to a further rise in cytosolic [Ca^2+^], causing inhibition of Cx43-hemichannel opening. Thus, it may be anticipated that activation of the actomyosin cytoskeleton may have dual function: (i) by acting as a “safety” mechanism to prevent excessive Cx43-hemichannel opening, which would be detrimental for the cell due to loss of ionic, metabolic and energetic gradients, and (ii) by serving as a “Ca^2+^-dependent” shutdown mechanism of Cx43 hemichannels, allowing a bimodal regulation of Cx43-hemichannel opening by cytosolic [Ca^2+^]. The Ca^2+^-dependent inhibition of Cx43 hemichannels promotes the occurrence of sustained Ca^2+^ oscillations, leading to increased survival [e.g., by increasing mitochondrial bio-energetics (Hajnoczky et al., 1995)] or controlling physiological functions like endothelial cell membrane permeability (De Bock et al., 2012a, 2013). In the absence of Ca^2+^-dependent inhibition of Cx43 hemichannels, the Ca^2+^-dependent opening of Cx43 hemichannels could lead to a sustained Ca^2+^ influx, causing intracellular Ca^2+^ overload and cell death.

Different mechanisms responsible for the negative regulation of Cx43 hemichannels by the actin/myosin contractile systems may be considered, including a direct or indirect link between Cx43 hemichannels and the actin/myosin cytoskeleton (Ponsaerts et al., 2012b). Unfortunately, while many cytoskeletal proteins have been shown to interact with Cx43, most, if not all, evidence has been obtained for Cx43 gap junctions (Herve et al., 2012). In that sense, it is interesting to note that increased contractility also negatively impacted Cx43-mediated gap junctional coupling in BCECs (D'hondt et al., 2007a). One of the underlying mechanisms proposed was the loss of tethering forces essential for stabilizing the interactions of the transmembrane proteins of tight junctions and the subsequent disrupted barrier integrity (Garcia et al., 1996; Satpathy et al., 2004). Hence, sufficient level of tethering forces may facilitate the docking of hemichannels from adjacent cells to form and maintain gap junction channels. Another aspect proposed was that increased actin/myosin contractility could interfere with the interaction of Zona Occludens-1 (ZO-1) with Cx43, thereby affecting Cx43 gap junction assembly (Hunter et al., 2005; Rhett et al., 2011). Loss of Cx43/ZO-1 interaction has been shown to increase gap junction size (Hunter et al., 2005). This promoted the trafficking of and assembly of the “undocked” hemichannels from the perinexus into gap junctional plaques, thereby increasing gap junctional communication while decreasing hemichannel-mediated signaling (Rhett et al., 2011). In BCECs, the binding of ZO-1 to Cx43 hemichannels was not detectable. In addition, it seemed that the inhibition of hemichannel opening by thrombin could be overcome by both TAT-CT10 and TAT-CT10ΔI, which lacks the last Ile residue critical for binding the PDZ2 domain of ZO-1 (Giepmans and Moolenaar, 1998; Ponsaerts et al., 2010b). Furthermore, the loss of tethering forces and barrier integrity could definitely not account for an inhibition of Cx43-hemichannel activity, since such an effect may rather lead to an increase in the ratio of “undocked” Cx43 hemichannels over docked Cx43 gap junctions. While it is clear that actin contractility negatively impacts Cx43-hemichannel activity, proper cytoskeletal organization is definitely required for ATP release and subsequent intercellular Ca^2+^ signaling, since these phenomena are impaired upon cytoskeletal destabilization using cytochalasin D exposure (Cotrina et al., 1998).

### Cx43 hemichannel opening is controlled by intramolecular loop/tail interactions

Since Cx43 gap junctions are critically and dynamically controlled by intramolecular interactions between the C-terminal tail and the second part of the cytoplasmic loop (L2 region) (Duffy et al., 2002; Delmar et al., 2004; Seki et al., 2004; Hirst-Jensen et al., 2007) and since Cx43 hemichannels consists of the same protein building blocks as Cx43 gap junctions, it was plausible that Cx43-hemichannel opening too was influenced by loop/tail interactions and that the actin/myosin cytoskeleton could impact Cx43-hemichannel activity by interfering with loop/tail interactions. An important insight was provided by the application of cell-permeable peptides covering the last 10 amino acids of the C-terminal tail of Cx43 (TAT-CT10) (Ponsaerts et al., 2010b). These residues harbor two important functional domains: (i) the C-terminal Ile^382^, which serves as the binding site for the PDZ2 domain of ZO-1 (Giepmans and Moolenaar, 1998), and (ii) the Asp^378^ and Asp^379^ residues, which serve as residues involved in the interaction with the L2 domain (Hirst-Jensen et al., 2007). Strikingly, pre-incubation of BCECs with TAT-CT10 peptide completely prevented the thrombin-induced inhibition of Cx43 hemichannels (Ponsaerts et al., 2010b). On the one hand, a TAT-CT10 peptide but lacking the C-terminal Ile residue (TAT-CT10ΔI) remained capable of alleviating the thrombin-induced inhibition of Cx43-hemichannel activity, indicating that altered Cx43/ZO-1-complex formation was not involved in this process (Ponsaerts et al., 2010b). On the other hand, a TAT-CT10 version in which Asp^378^ and Asp^379^ have been mutated to Ala residues completely lost its ability to alleviate the thrombin-induced inhibition of Cx43-hemichannel activity (D'hondt et al., 2013b). Consistent with this, while biotinylated CT10 peptides immobilized to a streptavidin-coated sensor chip could bind the L2 and cytoplasmic loop region in surface plasmon resonance experiments, biotinylated CT10 peptides in which Asp^378^ and Asp^379^ were changed into Ala residues, completely lost this property (Ponsaerts et al., 2010b; D'hondt et al., 2013b). Similar findings were observed for Cx43 hemichannels ectopically expressed in HeLa and C6 glioma cells. Indeed, the inhibition of ectopically expressed Cx43 hemichannels by high cytosolic [Ca^2+^] could be alleviated by treatment of the cells with either TAT-CT10 or TAT-CT10ΔI but not with TAT-CT10^DD/AA^ (Ponsaerts et al., 2010b). Furthermore, the electroporation of a peptide covering the last 9 amino acids of the C-terminal tail of Cx43 (CT9) in MDCK cells completely abolished the occurrence of bradykinin-induced Ca^2+^ oscillations (De Bock et al., 2012b). This indicates that the loss of endogenous loop/tail interactions can occur during physiological Ca^2+^ signaling and is the underlying mechanism responsible for the Ca^2+^-dependent inhibition of Cx43 hemichannels.

Since the L2 region was identified as the target for the CT10 peptide, the effect of a cell-permeable L2 peptide on Cx43 hemichannel opening was also examined (Ponsaerts et al., 2010b). It was found that TAT-L2 prevented Cx43-hemichannel opening in BCECs induced by mechanical stimulation or by Ca^2+^-free solution (Figure 3). TAT-L2 also inhibited Cx43-hemichannel opening in Cx43-expressing HeLa/C6-glioma cells induced by moderate increases in cytosolic [Ca^2+^]. Interestingly, a mutant version of TAT-L2 (i.e., TAT-L2^H126K/I130N^) previously shown to be impaired for its ability to interact with the C-terminal tail (Seki et al., 2004), failed to inhibit the activity of endogenously and ectopically expressed Cx43 hemichannels (Ponsaerts et al., 2010b). In contrast, gap junctional coupling did not seem to be inhibited by TAT-L2. Hence, it is proposed that TAT-L2 by targeting the CT10 region of Cx43 hemichannels interferes with the occurrence of endogenous loop/tail interactions essential for Cx43-hemichannel activity. As such, the inhibitory effect of TAT-L2 could be prevented by co-application of TAT-CT10 (Ponsaerts et al., 2010b). Moreover, from these measurements and previous studies of the Delmar group (Seki et al., 2004), it also became clear that L2 oppositely affects Cx43 gap junctions, namely favoring their open state, while preventing/inhibiting Cx43 hemichannel opening. These findings were supported by the fact that Cx43^M239^-based hemichannels, which lack the complete C-terminal tail, fail to open. In contrast, Cx43^M239^-based gap junctions are active and remain open, even during conditions of acidification (Moreno et al., 2002). Therefore, it was anticipated that loop/tail interactions present in Cx43 gap junctions also exist in Cx43 hemichannels but with a functional outcome. To scrutinize this concept, it was shown that TAT-CT10 and TAT-CT10ΔI, but not TAT-CT10^DD/AA^, could restore Cx43^M239^-hemichannel activity (Ponsaerts et al., 2010b). It is important to note that the addition of TAT-CT10 is not sufficient to open Cx43 hemichannels, but rather facilitates their opening in response to triggers like voltage steps to positive membrane potentials or increased cytosolic [Ca^2+^]. Hence, our model proposes that loop/tail interactions are required to bring Cx43 hemichannel in a “ready to open” state (Wang et al., 2013a).

Further studies revealed a stretch of 9 amino acids within the L2 region of the cytoplasmic loop as the target of CT10 (Wang et al., 2013b). This Lys-rich stretch of amino acids is mainly positively charged, thereby allowing electrostatic interactions with the two negatively charged Asp residues within the CT10 region. Strikingly, a nonapeptide, called Gap19, displayed similar properties as TAT-L2, thereby causing inhibition of Cx43 hemichannels but not Cx43 gap junctions (Wang et al., 2013b). Gap19 naturally displayed strong cell-permeable properties, likely due to the Lys-rich stretch, thereby resembling the positively charged residues in the TAT cell-penetrating sequence. Consistent with this, Gap19 completely mimicked the actions of TAT-L2, thereby inhibiting the opening of Cx43 hemichannels in response to low extracellular [Ca^2+^] or moderate rises in cytosolic [Ca^2+^] (Wang et al., 2013b). The inhibitory effect of Gap19 was also analyzed at the electrophysiological level using whole cell patch clamp experiments, allowing the characterization of its effect on Cx43 hemichannel unitary currents (Wang et al., 2013a,b). Gap19 decreased the frequency of single Cx43-hemichannel openings, thereby reducing the open probability of the Cx43 hemichannels. Interestingly, Gap19 was also found to inhibit native Cx43 hemichannels present in ventricular cardiomyocytes, acutely isolated from pig hearts. The inhibition seems to be due to a right-shift of about 30 mV in the voltage-dependent opening of Cx43 hemichannels.

Finally, it is anticipated that the loss of loop/tail interactions essential for Cx43-hemichannel activity may underlie the inhibition of Cx43-hemichannel activity in response to increased actin/myosin contractility. Indeed, Cx43^M239^-based hemichannels fail to open, even in response of the myosin II ATPase inhibitor, (-)-blebbistatin (Ponsaerts et al., 2010b).

### Targeting loop/tail interactions in Cx43: selective Cx43-hemichannel inhibitors as novel tools in cell biology, physiology and pathophysiology

The concept of loop/tail interactions differentially controlling Cx43 gap junctions vs. hemichannels has spurred the development of peptide tools, including TAT-L2 and Gap19, to selectively inhibit Cx43 hemichannels while unaffecting Cx43 gap junctions in a variety of primary cell systems and tissues (Evans et al., 2012; Iyyathurai et al., 2013; Wang et al., 2013a). As such, these tools supplement knockdown/knockout approaches, which do not allow discriminating between gap junctions and hemichannels. Furthermore, these tools are not only selective for Cx43 hemichannels vs. gap junctions, but are also unlikely to target other Cx or Panx isoforms. In particular, the L2 region of Cx43 is very divergent among different Cx isoforms and the region that is targeted by L2 or Gap19, i.e., the last 10 amino acids of Cx43, is not present in other Cx isoforms (see Table 2 in Iyyathurai et al., 2013 for the BLAST results obtained by using the L2 and CT10 sequences of human Cx43 as sources). This is supported by experimental evidence, showing that Gap19 did neither inhibit Cx40 hemichannels nor Panx1 channels (Wang et al., 2013b).

These tools now set the stage for the discovery of novel cell biological and physiological roles for Cx43 hemichannels and the inhibition of pathological, excessive Cx43 hemichannel openings in disease conditions, including ischemia/reperfusion in the heart (Iyyathurai et al., 2013; Saez and Leybaert, 2014). Furthermore, these tools not only target plasmalemmal Cx43 hemichannels, but may also target Cx43 hemichannels present in the inner mitochondrial membrane (Boengler et al., 2013). For instance, Cx43 has been found in the inner mitochondrial membrane of subsarcolemmal mitochondria from ventricular cardiomyocytes (Boengler et al., 2009). Cx43 has been implicated in mitochondrial K^+^ uptake (Miro-Casas et al., 2009). Genetic ablation of Cx43 as well as Gap19 decreased the rate of mitochondrial K^+^ uptake in subsarcolemmal mitochondria (Boengler et al., 2013). Hence, the acute inhibition of Cx43 hemichannels using Gap19 elucidated a novel cell biological role for Cx43 hemichannels at the level of the mitochondria modulating mitochondrial K^+^ uptake.

TAT-L2 has also been applied in the context of the brain. In particular, micro-injection of the TAT-L2 peptide in the basolateral amygdala, a region of the brain involved in memory consolidation, of auditory fear-conditioned rats resulted in ablation of memory consolidation without impacting short-term memory, locomotion, or shock reactivity or without affecting synaptic transmission or interastrocyte gap junctional communication (Stehberg et al., 2012). The amnesic effect of TAT-L2 was related to an impaired gliotransmitter release from astrocytes, since a cocktail of gliotransmitters including glutamate, glutamine, lactate, D-serine, glycine, and ATP could restore the learning capacity of the rats. As such, TAT-L2 as a selective Cx43-hemichannel inhibitor led to the discovery of a novel physiological role for Cx43 hemichannels as release pathways for gliotransmitters essential for memory consolidation in the basolateral amygdala.

TAT-CT10 has been applied in the context of satellite glial cells, the main glia in sensory ganglia. They are proposed to prevent the formation of electrical and chemical synapses between neighboring neurons. However, paracrine signaling between glial cells and sensory neurons may occur. A role for hemichannels has been implicated in increased vagal nerve activity after nodose neuron exposure to Ca^2+^-free solution. Interestingly, TAT-CT10 displayed a similar effect as Ca^2+^-free solution on these sensory neurons, thereby facilitating paracrine signaling between satellite glial cells and neurons (Retamal et al., 2014).

Gap19 has been applied in the context of the heart exposed to ischemia/reperfusion (Wang et al., 2013b). Importantly, Gap19 not only inhibited the “physiological” opening of native Cx43 hemichannels present in ventricular cardiomyocytes obtained from healthy hearts, but also suppressed the excessive, “pathophysiological” opening of Cx43 hemichannels from ventricular cardiomyocytes exposed to metabolic inhibition triggered by a mitochondrial uncoupler and a glycolysis inhibitor (Wang et al., 2013b). Consistent with this, Gap19 also suppressed the deleterious effects of ischemia/reperfusion on cardiomyocyte viability *in vitro*, thereby maintaining the cell volume of cardiomyocytes and limiting the occurrence of cell death. Finally, *in vivo* ischemia/reperfusion experiments in mice showed that pre-incubation with Gap19 significantly decreased the infarct size.

## Remaining questions and future directions

### The link between the actin/myosin cytoskeleton and Cx43 hemichannels?

Two models for the regulation of Cx43 hemichannels by actin/myosin contractility, including a “direct” linker protein that bridges the C-terminal tail of Cx43 with the actin/myosin cytoskeleton and an “indirect” membrane-embedded “sensor” of the contractile state of the actin/myosin cytoskeleton that can also bind to the C-terminal tail of Cx43 hemichannels via an adaptor protein (Ponsaerts et al., 2012b). Different proteins associated with the actin/myosin cytoskeleton have been implicated in interactions with Cx43 proteins, but also actin may directly bind Cx43 proteins (Butkevich et al., 2004; Li et al., 2005; Wall et al., 2007; Sin et al., 2009; Vitale et al., 2009; Herve et al., 2012). In addition, we may not exclude that these interactions could be modulated by Ca^2+^-dependent changes in the phosphorylation state of Cx43 hemichannels due to the activation of Ca^2+^-dependent kinases or phosphatases (Solan and Lampe, 2009; O'quinn et al., 2011; Palatinus et al., 2011; Marquez-Rosado et al., 2012).

### Other domains within the C-terminal tail contributing to the control of Cx43-hemichannel activity?

The current model for loop/tail interactions controlling Cx43-hemichannel activity involves the Gap19 region in the cytosplasmic loop and the CT10 region in the C-terminal tail. Although these domains are sufficient to either inhibit Cx43-hemichannel opening or to restore Cx43-hemichannel activity, it cannot be excluded that other domains in the C-terminal tail of Cx43 can modulate or contribute to the interactions with the Gap19/L2 region of the cytoplasmic loop and thus the activity of Cx43 hemichannels. In particular, previous studies in Cx43 gap junctions have implicated other domains acting as the gating particle controlling Cx43 gap junction activity. For instance, a 17-mer peptide corresponding to the region 271 to 287 of Cx43 is able to inhibit Cx43 gap junctions (Calero et al., 1998). However, the relevance of this region and/or other regions within the C-terminal tail for controlling Cx43-hemichannel activity remains to be elucidated.

### Loop/tail interactions as a general concept in controlling the activity of other Cx isoforms?

It also remains to be elucidated whether the concept of loop/tail interactions being critical for the activity of Cx hemichannels is limited to Cx43 or can be a general mechanism operative in other Cx isoforms. In any case, although the “hot spots” for loop/tail interactions in Cx43 seem not highly conserved among other Cx isoforms (see Iyyathurai et al., 2013 for a detailed discussion), it could be possible that other amino acid stretches or motifs within the loop and tail of other Cx isoforms may establish such interactions. For instance, a recent report from Jiang and co-workers indicates that loop/tail interactions may exist in Cx46 hemichannels (Ren et al., 2013). Indeed, the Cx46^G143R^ mutation decreases gap junctional coupling while increasing hemichannel activity. Consistent with this, a GST-fusion protein containing the Cx46 loop region displayed enhanced Cx46-binding properties when containing the cataract-causing G143R mutation (Ren et al., 2013). These data seem to suggest that at least for Cx isoforms belonging to the gap junction family, loop/tail interaction may be operative and control the opening of hemichannels. Interestingly, the introduction of an additional positive charge in the loop enhancing Cx46-hemichannel activity and promoting Cx46 binding seems to suggest a prominent role for charge-based interactions, similar to Cx43. Until now, there is no evidence whether other Cx isoforms belonging to other gap junction families can be controlled by loop/tail interactions. Of note, Cx32 also displays a bell-shaped dependence toward cytosolic [Ca^2+^] rises (De Vuyst et al., 2006), but it remains to be established whether this is due to the occurrence of loop/tail interactions and their modulation by contractility.

## Conclusions

We hereby show that primary BCECs are a good model system for studying the properties of native Cx43 hemichannels and their regulation by physiological signaling events. These studies ought to be complemented with knockdown experiments and ectopically expressed Cx43 hemichannels, allowing the expression of mutated and truncated Cx43 versions. The integrated approach has led to important discoveries, including the negative regulation of Cx43-hemichannel opening by the actin/myosin contractile system, the essential role of loop/tail interactions for Cx43-hemichannel activity and to the development of novel peptide tools that allow selective Cx43-hemichannel inhibition without affecting Cx43 gap junctions or hemichannels formed by other Cx isoforms.

### Conflict of interest statement

The authors declare that the research was conducted in the absence of any commercial or financial relationships that could be construed as a potential conflict of interest.
